# Health, Climate Change and Energy Vulnerability: A Retrospective Assessment of Strategic Health Authority Policy and Practice in England

**DOI:** 10.4137/EHI.S950

**Published:** 2008-11-17

**Authors:** J. Richardson, F. Kagawa, A. Nichols

**Affiliations:** 1 Professor of Health Services Research, Faculty of Health and Social Work, University of Plymouth, 3 Portland Villas, Drake Circus, Plymouth, Devon PL4 8AA, U.K.; 2 Research Team Coordinator. Centre for Sustainable Futures, University of Plymouth, Drake Circus, Plymouth, Devon PL4 8AA, U.K.; 3 Faculty of Health and Social Work, University of Plymouth, 3 Portland Villas, Drake Circus, Plymouth, Devon PL4 8AA, U.K.

**Keywords:** sustainability, climate change, health

## Abstract

**Background:**

A number of policy documents suggest that health services should be taking climate change and sustainability seriously and recommendations have been made to mitigate and adapt to the challenges health care providers will face. Actions include, for example, moving towards locally sourced food supplies, reducing waste, energy consumption and travel, and including sustainability in policies and strategies. A Strategic Health Authority (SHA) is part of the National Health Service (NHS) in England. They are responsible for developing strategies for the local health services and ensuring high-quality performance. They manage the NHS locally and are a key link between the U.K. Department of Health and the NHS. They also ensure that national priorities are integrated into local plans. Thus they are in a key position to influence policies and practices to mitigate and adapt to the impact of climate change and promote sustainability.

**Aim:**

The aim of this study was to review publicly available documents produced by Strategic Health Authorities (SHA) to assess the extent to which current activity and planning locally takes into consideration climate change and energy vulnerability.

**Methods:**

A retrospective thematic content analysis of publicly available materials was undertaken by two researchers over a six month period in 2008. These materials were obtained from the websites of the 10 SHAs in England. Materials included annual reports, plans, policies and strategy documents.

**Results:**

Of the 10 SHAs searched, 4 were found to have an absence of content related to climate change and sustainability. Of the remaining 6 SHAs that did include content related to climate change and energy vulnerability on their websites consistent themes were seen to emerge. These included commitment to a regional sustainability framework in collaboration with other agencies in the pursuit and promotion of sustainable development.

Results indicate that many SHAs in England have yet to embrace sustainability, or to integrate preparations for climate change and energy vulnerability within their organisational strategies. Evidence also suggests that SHAs that have recognised the importance of sustainability within their documentation and policies have yet to fully demonstrate this in practice through the implementation of these policies.

**Conclusions:**

Further research is required to investigate means by which SHAs (U.K.) and agencies responsible for health service policy in other countries may be enabled to include a greater consideration of sustainability and climate change within their policies, and to find effective ways of implementing these policies within daily working practice.

## Introduction

Climate change and energy vulnerability present significant challenges to the ability of populations to maintain health and deliver services to the sick. The adverse effects are likely to be particularly directed at the poor as energy poverty will increase and food security will be compromised, thus increasing inequalities in health (Haines et al. 2006; McMichael et al. 2006).

McMichael et al. (2006) review the published estimates of future health effects of climate change and present a schematic summary of the main pathways by which climate change affects health. Significantly the authors differentiate between climate-health relationships that are easy to define (physical hazards due to heatwave, flooding, storms, fires and infectious disease), and those that are less easy to quantify (disruption to regional food supplies and fisheries, population displacement, loss of livelihoods), but may have detrimental effects including risks of malnutrition and mental health problems. Earlier assessments of the potential effects of climate change (Haines and Patz, 2004) show similar pathways and emphasise the need for mitigation and adaptation measures (Haines et al. 2006). Furthermore, the need for countries to undertake national assessments of the impacts of climate change on human health was noted as early as 1999 (Kovats et al.).

In the U.K. an increase in mean annual temperature of 2.5–3 degrees centigrade by the end of this century is likely to have a number of consequences. Increase in flooding will result in loss of life, injuries, water contamination and restrictions, loss of homes (temporary accommodation), and potential problems with businesses and employment. Excessive rainfall will lead to bacteria in surface water due to run-off from agricultural land. Increased water temperature is likely to damage fish-stocks, increase algal blooms in reservoirs with consequent decreased efficiency in the chemical coagulation in drinking water. Increased heat will result in drought and disruption to water supplies, frequent and long heat waves resulting in dehydration and deaths, increase in air pollution resulting in respiratory problems, increase in skin cancers, and increasing incidence of food-borne diseases. Though malaria is unlikely to become a problem in the U.K. vector-borne diseases such as Lyme Disease may become more prevalent. (Source: Health Effects of Climate Change in the U.K.: An update of the Department of Health report 2001/2002, Department of Health and Health Protection Agency). The consequences will be higher demand on emergency and health services, and rising mental health problems. Add to this the challenges of meeting local healthcare needs in a post peak-oil scenario, with possible limited access to some medicines, transport and energy difficulties, and we could be facing a public health disaster (Frumkin et al. 2007).

Haines et al. (2006) emphasises the need for Public Health strategies such as improved surveillance in order to adapt to the impact of climate change, as well as advocating action to mitigate climate change by reducing the use of fossil fuels. With its large estate, purchasing power, and significant numbers of employees (more than 1.3 m people) the U.K. National Health Service (NHS) has enormous power to mitigate the impact of climate change by implementing sustainable practices and encouraging its employees to do so (Coote, 2006).

So what is the NHS doing about this? NHS Trusts have emergency plans in place to deal with disasters such as major incidents. Increasingly these will require plans for events such as the flash flooding we have seen recently in the U.K. Future public health planning will need to take account of the prediction of possible scenarios and will inevitably involve multi-agency working and detailed assessment of local resources. The Department of Health has produced a heatwave plan which is deployed locally, and together with the Health Protection Agency has produced a consultation document on the health effects of climate change in the U.K.

One initiative aimed at enabling people and organisations in the NHS and wider community to engage in activities that support sustainability is ‘The Convergence of Health and Sustainable Development’. This is a network of health and other professionals committed to promoting sustainable development within the NHS. This network has produced a Manifesto, adopting the Brundtland definition, defining sustainable development as ‘the integration of environmental, social, political and economic development, underpinned by equity. It meets the needs of the present without compromising the ability of future generations to meet their own needs’. This Manifesto proposes a commitment to promoting sustainable development in the NHS and wider community and strengthening the position of sustainable development within the NHS workforce. Signatories to the manifesto include the Faculty of Public Health of the Royal Colleges of Physicians of the U.K. Scottish Environmental Protection Agency, the U.K. Public Health Association, as well as a number of senior public health academics and NHS managers.

It has been suggested that the responsibility of healthcare practitioners to protect and promote the health of the public should be extended to working to prevent climate change (Gill et al. 2007). The U.K. Public Health Association (2007) outline strategies for promoting health and sustainable development, and the Climate and Health Council has been established as a not-for-profit international organisation aimed at mobilising health professionals across the world to take action to limit climate change and its effects on human health (http://www.climateandhealth.org/). The U.K. Faculty of Public Health (2008) recently published a document outlining the action that can be taken at an organisational and individual level. This was closely followed by the publication of the U.K. Department of Health Guidance Document on The Health Impact of Climate Change: Promoting Sustainable Communities (April 2008). The British Medical Journal has set up a carbon council aimed at ‘harnessing the intelligence and imagination of health professionals to expedite the transition to a low carbon world’ (Stott, 2006), and efforts are being made to reduce the carbon footprint of attending medical conferences (Roberts and Godlee, 2007).

Whilst the potential effects of climate change and energy efficiency are being considered by a number of public health agencies, little is known about current efforts to plan for these effects in a strategic and practical way at a more local level. The Sustainable Development Commission NHS Good Corporate Citizenship guidance (http://www.corporatecitizen.nhs.uk/) describes how NHS organisations can embrace sustainable development and tackle health inequalities through their day-to-day activities. This self-assessment model helps organisations to identify and assess their contribution to good corporate citizenship and suggests ideas for future action, providing guidance on transport, procurement, facilities, management and new buildings. Examples of action include sourcing healthy and locally produced food, and the development of Park and Ride schemes. More recently the U.K. NHS Sustainable Development Unit has launched a Draft Carbon Reduction Strategy (May 2008) that sets out a framework for NHS organisations in England to reduce their carbon emissions.

Other examples of good practice include the Gwent Healthcare NHS Trust 25-year performance contract with energy services company, Honey-well, which has enabled the introduction of dual fuel burners, a Combined Heat and Power plant and seen the rollout of water conservation measures across the organisation (http://www.healthexec.tv/cgi-bin/details.pl?action=pre&id=426).

Given the emphasis at National level and the increasing good examples to draw upon, Strategic Health Authorities are well placed to provide direction on sustainability and efforts to mitigate and adapt to the effects of climate change. A Strategic Health Authority (SHA) is part of the U.K. National Health Service (NHS) in England. They are responsible for developing strategies for the local health services and ensuring high-quality performance. They manage the NHS locally and are a key link between the Department of Health and the NHS. They also ensure that national priorities are integrated into local plans (http://www.osha.nhs.uk/).

The aim of this study was to review publicly available documents from the ten Strategic Health Authorities in England and to assess the extent to which their current activity and future/strategic planning takes into consideration climate change and energy vulnerability.

## Methods

A retrospective thematic content analysis of publicly available materials was undertaken over a 6 month period. The websites of all 10 Strategic Health Authorities (SHAs) in England (U.K.) were searched. Materials obtained from these searches typically consisted of the most recent annual reports and plans of the SHAs. These materials were available to the public under the requirements of the Freedom of Information Act (2000); consequently ethical approval for this research was neither required nor sought.

Website searches were carried out by two researchers, each researcher was allocated 5 SHA websites to investigate. All members of the research team contributed to and agreed upon the construction of the coding system (Appendix 1) used during the website searches. This coding system consisted of 3 main codes addressing sustainability, energy vulnerability and climate change. Each of these main codes contained a variety of relevant sub-codes and considered mitigation and adaptation measures. Examples of text found within SHA documents were allocated to appropriate codes within the coding system. Reliability testing was achieved by members of the research team independently reviewing and re-coding a selection of material previously coded by their colleagues.

An exploratory, thematic content analysis of the data was employed and will be used to inform the discussion of themes, notions and concepts arising from the data.

Methods such as those outlined above have previously been used successfully, and have been advocated in the literature (Yin, 1994; Watkins et al. 2006; Reason and Garcia, 2007). For example, Yin (1994) argues that the analysis of evidence obtained from the searching of documentation can aid attention to detail, while Reason and Garcia (2007) find that smaller data sets comparable to those obtained during the searches of the SHA websites may well be suited to the kind of qualitative thematic content analysis described above.

## Results

Of the 10 SHA websites searched, 4 were found to have an absence of content related to climate change and sustainability. Of the remaining 6 SHAs that did include content related to climate change and sustainability on their websites, consistent themes were seen. Firstly, it was clear that SHAs that had discussed and considered climate change and sustainability recognised that wider influences and multiple determinants would be involved in achieving sustainability and managing climate change. For example, SHA 6 stated that:

Environmental damage such as air pollution; poor water and lack of water; contaminated food or lack of food; poorly designed buildings; heavy road traffic and places built so that people cannot exercise are all bad for health and cause environmental damage. Pursuit of sustainable development can profoundly influence population health and the implementation of preventative health measures (and) can help attain the goals of sustainable development.

Similar recognition of multiple determinants was provided by evidence obtained from the web-sites of SHA 8 and SHA 3 that also emphasised the importance of individuals. It was proposed that staff awareness of their own carbon footprint may have a positive impact on sustainability, and contribute to reductions in personal contributions to climate change.

SHA 10 and SHA 5 acknowledged the multiple factors that may contribute to climate change and have an adverse impact upon sustainability. In response to this, documents found on their websites made positive statements in regard the prioritisation of efforts related to sustainability through the efficient use of fuel, estates and services. For example:

The SHA is also looking at an approach by the NHS to sustainable development. This places importance on having a high level objective for the NHS in relation to carbon reduction and identifying short and long term priorities. The SHA recognises the need for detailed work at a regional and local level on energy efficiency, sustainable buildings, transport and procurement.

Furthermore, SHA 10 and SHA 3 were actively engaging with the broader communities which they served and in partnership with other organisations with similar goals. For example:

We will encourage any large-scale health project to take account of the environment when approving new buildings, and we are working with ****shire Forward and the Carbon Trust to make sure these projects work in line with ‘green’ guidelines.

This willingness to work in partnership with other organisations was echoed by SHA 6 and their commitment to a regional sustainability framework in collaboration with other agencies in the pursuit and promotion of sustainable development.

A readiness to take part in such collaborative projects could be seen as evidence of SHAs acknowledging their role, and making explicit their policies and strategies in regard to sustainability. For example, SHA 5 claimed that they were aware that:

We need to act as a role model for other NHS organisations and have begun to put in place policies that tackle some of the sustainability and environmental issues that face us all today.

Furthermore SHA 5 was seen to express commitment to corporate citizenship and contributing to the wellbeing and sustainability of the population which they served. Similarly, SHA 3 was clear that they were responsible:

For making recommendations to the board on the corporate social responsibility strategies (environmental, social, ethical, and sustainable development) of the authority in discharging its responsibilities.

And for:

Building a sustainable and affordable health service that meets the challenges of the future.

The SHAs’ commitments to achieving sustainability and combating climate change were seen to be reflected in their strategies and policies. Of the 10 SHA websites included in the study, 6 were found to contain evidence of strategies and policies related to sustainability and climate change. However, of these 6 only 2 were found to contain evidence of these policies and strategies being translated into tangible action. For example SHA 10 was seen to have actively sought to make its NHS buildings more environmentally friendly by attaching windmills to them in order to generate power. Another example was provided by SHA 5 which printed its annual report on recycled paper and was committed to using locally sourced materials.

## Discussion

The above evidence indicates that a number of SHAs have recognised the impacts climate change may have on the health of the populations which they serve, and have responded by making commitments to implement policies in regard to sustainable development and other measures to combat climate change. However, limited evidence was found of strategies that assess and plan for energy vulnerability. Perhaps the most notable result of the website searches was the lack of content related to actual action being undertaken. The evidence we have found indicates that Strategic Health Authorities have failed to develop local strategies and plan action to mitigate and adapt to climate change and sustainability, and that national priorities set out in DH guidance have yet to be integrated into SHA policy and planning. However we recognise that responses to recent U.K. DH policy will not necessarily be forthcoming in Annual Reports published some months ago.

Of particular concern is the absence of any references to climate change and sustainability on 4 of the 10 SHA websites. Although direct U.K. Government (Department of Health) policy on health and sustainability is fairly recent, concerns about the consequences of climate change for health and health care have been published in the medical press for a number of years now. This is a global problem that will require both global and local action, and agencies responsible for health and environmental strategy will be required to take the lead on the development and implementation of appropriate policy.

Population structure and density, and poverty are relevant to climate change, as the elderly, sick and the poor are more vulnerable to weather extremes such as heatwaves, storms and floods (Confalonieri et al. 2007; Millennium Ecosystem Assessment). However it was not possible to assess the extent to which SHAs took account of vulnerability and equity when developing their sustainability strategies.

It could be suggested that an ‘ideal’ SHA policy would include the following: Mitigation measures to reduce carbon emissions and energy use. For example implementing procurement policies for energy efficient buildings, reducing travel by promoting tele/web and video-conferencing (U.K. NHS Sustainable Development Unity has launched a Draft Carbon Reduction Strategy May 2008). Regarding policy recommendations for mitigation, the UN Human Development Report (UNHDR) includes recommendations for energy efficiency and use of renewable energy.

Adaptation measures to address potential problems through policies to increase surveillance and develop sustainable communities (U.K. Department of Health Guidance Document on The Health Impact of Climate Change: Promoting Sustainable Communities April 2008). Focused action to develop resilience to empower and enable vulnerable people to adapt to climate change through investments in social protection, health, education and other measures (UNHDR). Initiatives such as the Transition Town movement that aim to build resilience in order to adapt to a low carbon economy and reduced availability of oil can also promote adaptation to climate change (see: Transition Town Network).

The development of joint-working and multi-agency strategies to proactively promote sustainability and meet the needs of emergencies arising from the impact of climate change and energy vulnerability. For example mobilising joint working to: address issues such as fuel poverty relief; develop multi-agency strategies to respond to flash flooding; work with schools and elderly care establishments to raise awareness about some of the challenges in a more proactive way; develop policies for working with the Meterological Office for forecasting and weather warnings.

Further research is required to investigate means by which SHAs (U.K.) and agencies responsible for health service policy in other countries may be enabled to include a greater consideration of sustainability, climate change and energy vulnerability within their policies, and to find effective ways of implementing these policies within their daily working practice.

## Appendix 1

**Table:** Criteria used to guide SHA website searches.

**Organisation**	**Annual Report**	**Annual Plan**	**Associated Documents**
Criteria			
Sustainability general			
Sustainability estates			
Sustainability transport			
Sustainability waste			
Sustainability planning			
Sustainability supplies and services			
Sustainability staff development			
Energy Vulnerability general			
Energy Vulnerability estates			
Energy Vulnerability transport			
Energy Vulnerability waste			
Energy Vulnerability planning			
Energy Vulnerability supplies and services			
Energy Vulnerability staff development			
Energy Vulnerability Multi-agency working			
Climate change infectious disease			
Climate change flooding			
Climate change disaster management			
Climate change Air quality/pollution			
Climate change Multi agency working e.g. Met office/Environment agency			
SHA/PCT links/refs to relevant sustainability documents.			
Evidence of these being acted upon?			
